# Society Meetings

**Published:** 1917-10

**Authors:** 


### SOCIETY MEETINGS

**Brief reports and announcements of meetings of societies of Western New York. and those of general scope, are requested from Secretaries. Copy should be on hand the fifteenth of the month. Full transactions will be published at cost of composition.**

**DOCTOR: Is your Society properly represented here? If not, please bring our standing announcement to the attention of the members.**

**The Eighth District Branch of the Medical Society of the State of N. Y.** held its 12th annual meeting in Alumni Hall of the University of Buffalo, Sept. 13 and 14. Beside the regular scientific and business sessions, clinics were held at the General and Municipal Hospitals and Laboratory Demonstrations at the State Institute for Study of Malignant Disease. As the full report will appear in the State Journal, we feel that it is only courteous to mention the meeting briefly.

**The Elmira Academy of Medicine** held its regular meeting in its rooms in the Federal Building, Elmira, Sept. 5. Program: Iritis, L. D. Mottram; Cardio-therapy of the Obese, C. W. Lieb; Use of the Fluoroscope, N. A. Soble. R. B. Howland, Pres.; O. J. Bowman, Sec.

---

**The Chemung County Medical Society** held a regular meeting Sept. 18 in its rooms in the Federal Building, Elmira. Dr. Harvey P. Jack of Hornell read a paper on Bladder Disease of the Aged; Dr. Henry J. Mulford of Buffalo, on the Mind of the Child. Dr. Clyde Carey, Pres.; Dr. Mabel Flood, Sec.

---

**The American Public Health Assn.** will hold a war meeting in Washington, Oct. 17-20, this meeting supplanting the announced annual meeting at New Orleans, Dec. 4-7. The papers will deal with food supply, communicable disease among soldiers, venereal diseases, the health of the civil population, all with special reference to war problems. Non-members will receive the preliminary program on application to the association at 128 Massachusetts Ave., Boston.

